# An Analytical View on the Use of Flucloxacillin for Outpatient Parenteral Antimicrobial Therapy

**DOI:** 10.3390/microorganisms12102039

**Published:** 2024-10-09

**Authors:** Tam Nguyen, Isabel Spriet, Charlotte Quintens, Lotte Vander Elst, Pham Thi Thanh Ha, Ann Van Schepdael, Erwin Adams

**Affiliations:** 1 Department of Pharmaceutical and Pharmacological Sciences, Pharmaceutical Analysis, KU Leuven, Herestraat 49, O&N2, PB 923, 3000 Leuven, Belgium; thiminhtam.nguyen@kuleuven.be (T.N.); ann.vanschepdael@kuleuven.be (A.V.S.); 2 Hospital Pharmacy Department, University Hospitals Leuven, Herestraat 49, 3000 Leuven, Belgium; isabel.spriet@uzleuven.be (I.S.); charlotte.quintens@uzleuven.be (C.Q.); lotte.vanderelst@uzleuven.be (L.V.E.); 3 Department of Analytical Chemistry and Drug Quality Control, Hanoi University of Pharmacy, 13–15 Le Thanh Tong, Hoan Kiem, Hanoi 100000, Vietnam; thanhha.pham@hup.edu.vn

**Keywords:** flucloxacillin, liquid chromatography (LC), outpatient parenteral antimicrobial therapy (OPAT), stability

## Abstract

Although the addition of buffers provides improved stability to flucloxacillin (FLU) solutions, unbuffered solutions are often preferred in clinical practice. The first purpose of this study was to investigate whether a 50 mg/mL solution of FLU in normal saline is stable for 24 h at 33 °C so that it can be applied for outpatient parenteral antimicrobial therapy (OPAT) using portable elastomeric infusion pumps (PEIPs). When the PEIPs were stored in an oven at 33 °C and deflated over 24 h, the volume of the collected solution, pH, and FLU concentration were checked every 4 h. Obtaining better results than expected based on the literature data, other storage conditions, such as refrigeration, room temperature (RT), 37 °C, refrigeration followed by 24 h at 33 °C and 37 °C, and different batches/brands, were also tested. This study confirmed the pronounced effect of temperature on the stability of FLU and also showed the relationship between the stability of FLU and the initial pH of the solution. FLU was quite stable at refrigeration and RT conditions, with more than 99% and 95% remaining. After 24 h at 33 °C, more than 92% of FLU was still present in the solution, while this number decreased to less than 85% when the storage temperature reached 37 °C. The remaining percentage was found to be even lower when the solution was stored at 2–8 °C for 6 days, followed by 24 h storage at 33 °C or 37 °C, with losses of 17% and 30%, respectively. The stability of FLU became worse when the initial pH of the solution was lower than 5.9 since the concentration of FLU dropped to less than 90% after 24 h at 33 °C, and a precipitate started to form when the initial pH of the solution was around 5.3. Therefore, FLU in PEIPs could be employed for 24 h if the temperature was ideally not more than 33 °C, while the pH should be not less than 5.9 upon reconstituting the FLU solution.

## 1. Introduction

Outpatient parenteral antimicrobial therapy (OPAT) refers to the administration of parenteral antimicrobial treatment in a minimum of two doses on separate days without the need for hospitalization [1]. OPAT is primarily indicated for patients requiring long-term intravenous (IV) antibiotic therapy to treat complex respiratory tract infections, bone and joint infections, complicated urinary tract infections, and central nervous system infections. OPAT offers many potential benefits, including reduced risk of hospital-acquired infections, cost savings due to reduced bed use, and enhanced patient satisfaction [2,3]. However, it also raises a concern. In Europe, home care nurses have to visit the patients’ homes several times per day to prepare and administer each dose of antibiotics [4]. This can lead to some inconvenience for both healthcare professionals and patients. The use of portable elastomeric infusion pumps (PEIPs), which are non-electric pumps consisting of an elastomeric balloon to release the drug solution at a constant flow, could be an ideal solution for continuous administration of antibiotics during 24 h. In this way, nurse visits can be reduced to once a day. However, the antibiotics should be stable during the administration period. The structure of the antibiotic, temperature, and duration of the storage, combined with the drug concentration, diluents, and container composition, are the main factors affecting the drug stability [2]. For instance, beta-lactam antibiotics are susceptible to degradation due to the instability of the beta-lactam ring. Furthermore, it was already shown that the temperature of antibiotic solutions in a PEIP could be higher than the standard room temperature if the patient wore the PEIP around the hip or put it under the blankets at night. So, it was found that the temperature of the pouch reached up to 33 °C [5].

**Figure 1:**
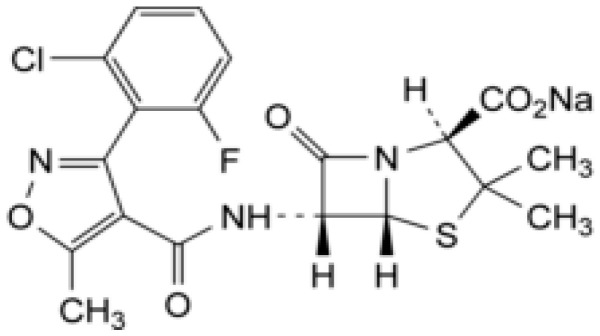
Chemical structure of flucloxacillin sodium [9].

Flucloxacillin (FLU) is a beta-lactam antibiotic belonging to the penicillin class. It is active against Gram-positive bacteria, including β-lactamase producing staphylococci, by inhibiting the cell wall biosynthesis of the bacteria. It is used to treat various infections, with a particular focus on skin and soft tissue and respiratory infections [6,7,8]. There are two forms of FLU, including FLU magnesium octahydrate and FLU sodium (Figure 1), the latter being the most commonly used in pharmaceutical formulations [9].

Because of the beta-lactam ring, FLU is relatively unstable in aqueous solutions. Several stability studies have been published. Voumard et al. [5] conducted a stability study of FLU prepared in normal saline at a concentration of 33 mg/mL under real-life conditions, i.e., the volunteer wore the elastomeric pump and had normal daily activities. Carroll [10] prepared FLU in saline to obtain 50 mg/mL solutions and stored them at 2–8 °C for 6 days. Next, the elastomeric infusion devices were kept at 31 °C for 24 h. PEIPs were also stored at 31 °C during 17 h and then 7 h at 37 °C. To et al. [11] also refrigerated the FLU solutions at 2–8 °C for 6 days before incubating them at 37 °C for 24 h. The authors tested two different concentration solutions, 50 mg/mL and 120 mg/mL, using several diluents (i.e., 0.9% NaCl, water for injection, and phosphate buffer). Buffered solvent was also employed by Allwood et al. [12]. The authors dissolved FLU in 0.3% *w*/*v* citrate-buffered saline pH 7.0 to obtain two concentration levels, 10 mg/mL and 50 mg/mL. The stability of the solutions was tested after 14 days, including 13 days stored at 2–8 °C, followed by 24 h at 32 °C. All these publications focused on chemical stability, i.e., the FLU concentration remaining after storage, first in the refrigerator, followed by higher temperatures. FLU degradation varied from 6% to 60% and was highly dependent on storage conditions. On the other hand, physical aspects, including changes in solution color, clarity, and pH, were hardly described, and a systematic overview is lacking.

In some countries, portable infusion pumps are filled and then stored in the refrigerator for several days before use. To improve the stability, sometimes a buffer is added [12,13]. However, this is not common practice. At the University Hospitals Leuven (UZ Leuven), preference is given to unbuffered saline solutions of FLU, reconstituted right before administration without storage in the refrigerator.

Current patients may need a treatment of up to 12 g of FLU per day, usually spread over two doses of 6 g [11]. As a result, two visits of a nurse per day are needed, which is not ideal from an economic point of view and the patient’s comfort.

This study aimed to check whether IV solutions of 12 g FLU can be administered in OPAT via a 24-hour infusion with PEIP requiring only a once-daily visit of the home care nurse. This can be acquired if the concentration of FLU in the IV solution after 24 h of administration remains at least 90% of the starting concentration. A stability study of FLU solutions prepared in saline was conducted at a FLU concentration of 50 mg/mL (i.e., 12 g in 240 mL) and stored in PEIPs at 33 °C for 24 h. The stability of FLU solutions under these conditions has not been studied before. Besides the concentration of FLU, the pH, color, and clarity of the solution were evaluated. Although experimental conditions were different, the outcome was not completely coherent with data from the literature [10,11,12]. So, additional stability studies of FLU for 24 h at 37 °C were compared with those obtained at 33 °C. In addition, the stability of FLU for 6 days in the refrigerator followed by 24 h at 33 °C was performed, and the stability of FLU was also compared between different brands and batches. To determine the concentration of FLU while degradation products were rising over time, the stability-indicating LC-UV method was optimized and validated.

## 2. Materials and Methods

### 2.1. Reagents and Solvents

Floxapen vials (batches 1P49HK, 1P50HK, 2L26HK and FL0222027A) and Flucloxacillin Fresenius Kabi 2000 mg (batch 18Z2229) containing FLU sodium (equivalent to 2 g of FLU) were obtained from Aurobindo (Jette, Belgium) and Fresenius Kabi (Schelle, Belgium). FLU sodium CRS (batch 9) with a purity of 95.3% was bought from EDQM (Strasbourg, France). FLU working standard (WS) containing FLU sodium with a purity of 94.4% was established by comparing it with FLU sodium CRS. Sodium chloride 0.9% bags (250 mL) were obtained from Baxter (Eigenbrakel, Belgium). Potassium dihydrogen phosphate was purchased from Merck (Darmstadt, Germany). Sodium hydroxide and acetonitrile (ACN) were from VWR Chemicals (Radnor, PA, USA) and Thermo Scientific (Waltham, MA, USA), respectively. Water was purified by a Milli-Q water purification system from Millipore (Bedford, MA, USA).

### 2.2. Instruments and Materials

The type of PEIP used was a FOLFusor LV10 from Baxter (Eigenbrakel, Belgium) with a nominal volume of 240 mL, a maximum volume of 300 mL, and a nominal flow rate of 10 mL/h. The oven to maintain the temperature of the PEIPs was purchased from Memmert (Schwabach, Germany). The pH meter was supplied by Metrohm (Herisau, Switzerland). The HPLC system from Merck Hitachi (Darmstadt, Germany) consisted of an L-2130 pump, an automated sample injector L-2200, and an L-2450 UV detector. Data acquisition and processing were performed using EZChrom Elite software (version 3.1.6). All instruments were calibrated.

### 2.3. Liquid Chromatographic Conditions

Analyte separation was carried out on a Kinetex EVO-C18 column from Phenomenex (Torrance, CA, USA) (3 mm × 150 mm) with a particle size of 3.5 μm and a pore size of 100 Å. The mobile phase consisted of a mixture of mobile phase A (buffer pH 5.0—acetonitrile (72:28, *v*/*v*)) and mobile phase B (buffer pH 5.0—acetonitrile (50:50, *v*/*v*)). In the first 10 min, 100% of mobile phase A was delivered before decreasing gradually to 0% over 8 min. One min later, the gradient was returned to 100% of mobile phase A, and the system was re-equilibrated for 5 min before the next run. The buffer pH 5.0 was prepared by adjusting a 2.7 g/L solution of potassium dihydrogen phosphate to pH 5.0 with diluted sodium hydroxide solution (8.5 g in 100 mL). The flow rate and injection volume were 0.6 mL/min and 20 μL, respectively. The detector wavelength was set at 225 nm.

### 2.4. Liquid Chromatographic Method Validation

The developed method was validated in accordance with the ICH Q2 guideline [14], including selectivity, linearity, the limit of detection (LOD), the limit of quantification (LOQ), accuracy, and precision.

Selectivity: A blank solution (mobile phase A) and a placebo solution (sodium chloride 0.9%) were injected to make sure that they did not interfere with the principal peak. The selectivity was further evaluated by performing a forced degradation study using 0.01 M hydrochloric acid and 0.01 M sodium hydroxide. Degradation was also checked by heating the reference solution for 4 h at 60 °C;

Linearity: Linearity was assessed by establishing calibration curves of at least five concentrations ranging from 0.02 mg/mL to 0.07 mg/mL (equivalent to 40% to 140% of the injected FLU concentration) injected in duplicate. Determination coefficients (*r*^2^) should be no lower than 0.995, and residuals should be randomly distributed;

Sensitivity: The LOD and LOQ values were estimated at a signal-to-noise (S/N) ratio of 3 and 10, respectively;

Accuracy: Accuracy was evaluated by the recovery of known amounts from spiked solutions at three different concentrations. Triplicate injections of 0.04 mg/mL, 0.05 mg/mL, and 0.06 mg/mL (equivalent to 80%, 100%, and 120% of the injected FLU concentration) were analyzed. The recovery should be between 98% and 102%;

Precision: Intra-day precision was performed by consecutively injecting the 100% FLU solution (0.05 mg/mL) six times. Inter-day precision was performed on different days using two LC systems, columns, freshly prepared mobile phases, and reference and sample solutions. A relative standard deviation (RSD%) of not more than 2% should be obtained.

### 2.5. Stability Studies

#### 2.5.1. Stability of Flucloxacillin in Portable Elastomeric Infusion Pumps at 33 °C

- Preparation of portable elastomeric infusion pumps

Six Floxapen vials (batch 1P49HK) containing 2 g of FLU were used. The powder of each vial was dissolved in 20 mL of NaCl 0.9% using a syringe with a needle. Each vial was shaken until a clear and colorless solution was obtained. The solutions were then transferred into a measuring cylinder and further diluted with NaCl 0.9% to 240 mL, thus obtaining a FLU concentration of 50 mg/mL. Next, the solution was transferred to the PEIP via the fill port using a syringe with a needle. In total, four PEIPs were analyzed over two days. On a single day, two PEIPs were prepared at room temperature and then immediately placed in an oven at 33 °C for 24 h. The Luer Lock cap was removed, and the solution started flowing. The solution was collected into a conical flask covered with parafilm to avoid evaporation. Samples were taken every 4 h, implying a total of 6 samples per PEIP over 24 h. At each time point, two samples per PEIP were prepared by diluting with mobile phase A and injected in duplicate in the LC system. The reference solution was injected each time a new series of analyses was started.

- Sample analysis

The initial concentration of FLU was determined to make sure that nothing went wrong during the preparation of the pumps by comparing the peak areas of the PEIP solution with those of the reference solution, freshly prepared before use. Every 4 h, the solution was collected, and the collected solutions were weighed; the pH was measured, and the concentration of FLU was determined. In the end, the residual amount of solution in the PEIP was determined to check how much was not delivered. The initial concentration of each PEIP was taken as 100%, and the stability was then expressed as a percentage of this initial concentration, making it easier to compare the amount of degradation in each PEIP.

#### 2.5.2. Stability of Flucloxacillin in Solution at 37 °C

Two separate 50 mg/mL solutions of FLU were prepared from Floxapen vials (batch 1P49HK) and stored in an oven set at 37 °C. Every 4 h over 24 h, some aliquots were extracted from those solutions to check the color, clarity, pH, and concentration of FLU.

#### 2.5.3. Stability of Flucloxacillin in Solution under Refrigeration Conditions for 6 Days Followed by 24 h in an Oven

The content of 6 Floxapen vials (batch 1P49HK) was dissolved and diluted to obtain 240 mL of a solution with a concentration of 50 mg/mL of FLU. From this solution, 15 mL were used to determine the pH and initial concentration (0 h of day 0) before putting this aliquot in an oven at 33 °C. After 24 h, pH values and concentrations of FLU were checked. The remaining solution (225 mL) was transferred to an elastomeric pump and placed into the refrigerator for 6 days.

The exact time that the pump was put into the refrigerator on day 0 was recorded. Over 5 days, each day at the same time (0 h), the Luer Lock cap was opened to collect about 15 mL of the solution into a clean and dry conical flask. The solution was then kept at room temperature for 1 h before putting it into an oven at 33 °C. Each day (from day 0 to day 5), the pH and concentration of FLU were checked at 0 h and after 24 h of storage in the oven. Two PEIPs were prepared in this way. On day 6, the PEIPs were taken out of the refrigerator and kept at room temperature for 1 h. An amount of 15 mL of the solution from each PEIP was taken to determine the pH and FLU concentration at time 0 on day 6. The total volume of the solution taken per PEIP after 6 days was 90 mL. So, about 150 mL of the solution was left in each PEIP. PEIP 1 was then stored at 37 °C, while PEIP 2 was stored at 33 °C for 24 h. The Luer Lock caps were removed, and the PEIPs started deflating. The solutions were collected and used to check the pH and FLU concentration every 4 h. After 16 h, the PEIPs were empty, and no blockage of the PEIPs was observed. The collected solutions were used to determine the pH, color, turbidity, and FLU concentration. The remaining solutions were still kept in the oven for further determination at 20 h and 24 h. The initial concentration (at 0 h of day 0) was taken as 100%, and the stability was then expressed as a percentage of this initial concentration.

#### 2.5.4. Stability of Flucloxacillin from Different Batches and Brands

Due to the different initial conditions between our study and the papers from the literature, the (slightly) different properties of the tested products could be a factor that should be examined. Therefore, three other batches, 1P50HK, 2L26HK, and FL0222027A, from Aurobindo, and one batch, 18Z2229, from Fresenius Kabi, were tested. The solutions were prepared in the same way as described earlier to obtain a concentration of 50 mg/mL. The solutions were then stored at 33 °C for 24 h. The solution clarity, color, pH, and FLU concentration were determined at 0 h, 16 h, and 24 h.

#### 2.5.5. Stability of Flucloxacillin at Different Temperatures

Two solutions from Aurobindo vials (batches 1P50HK and FL0222027A) and one solution from Fresenius Kabi vials (batch 18Z2229) were prepared. Each solution was then divided into four portions and stored under four different conditions, including refrigerator, RT, 33 °C, and 37 °C. After 24 h storage, each solution was checked for clarity, color, pH, and FLU concentration.

## 3. Results

### 3.1. Method Development

**Figure 2:**
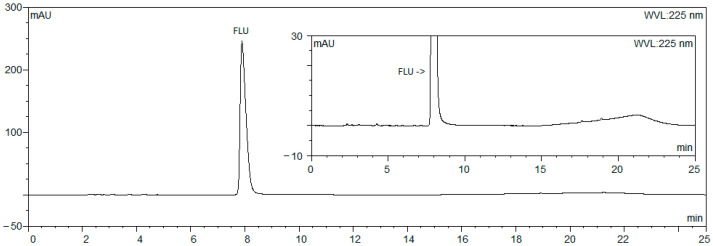
Chromatogram (with zoomed-in insert) obtained from sample solution in pump collected at 0 h.

**Figure 3:**
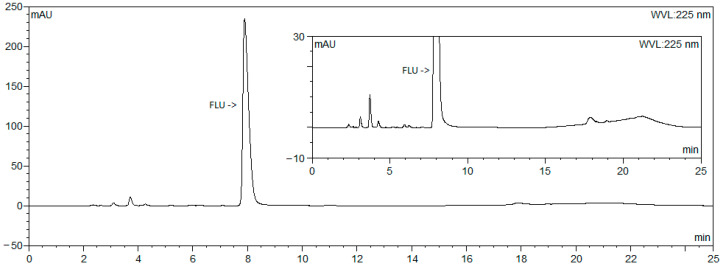
Chromatogram (with zoomed-in insert) obtained from sample solution in pump collected at 24 h.

The method for related substances from the FLU sodium monograph of the Ph. Eur., which was valid at the beginning of this study [9], was used as a starting point. Most of the impurities were eluted before the principal peak. The last impurity was eluted at 36 min. Therefore, the run time was too long, so it was not possible to follow up two PEIPs a day. Increasing the amount of organic modifier (ACN) in the mobile phase helped to reduce the run time, but the principal peak co-eluted with some impurities. As a result, a gradient program was introduced. This started with 28% of ACN since a higher percentage could not ensure the separation of the principal peak from the impurities. After the principal peak was eluted at around 8 min, the amount of ACN was increased so that the last impurity was eluted within 20 min. In addition, the system needs an extra 5 min to re-equilibrate so that one run is finished within 25 min. In this way, two PEIPs could be followed up each day. Two representative chromatograms are shown in Figure 2 and Figure 3.

### 3.2. Method Validation

**Figure 4:**
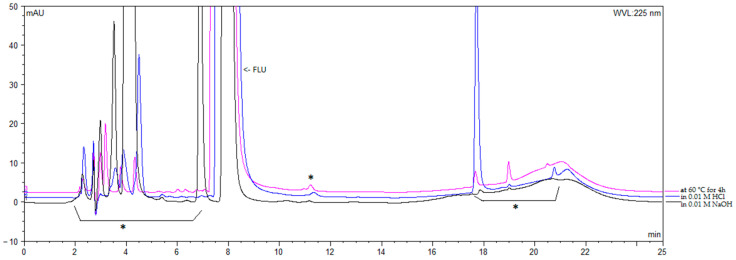
Overlaid chromatograms obtained from forced degradation solutions. The flucloxacillin (FLU) peak was eluted at 8 min. The peaks indicated with * correspond to degradation products.

Selectivity: The chromatograms of blank and placebo solutions did not show any peak at the retention time of FLU. The chromatograms obtained from forced degradation solutions showed that the main compound and the degradation products were well separated (Figure 4);

Linearity: The linear regression equation obtained was *y* = 1184.1*x* + 0.3 with *r*^2^ = 0.9998. Furthermore, residuals were randomly distributed, and the 95% confidence interval of the intercept included zero;

Sensitivity: The LOD value was measured at a S/N of 3 and amounted to 0.003 mg/mL. The LOQ value of 0.010 mg/mL gave a S/N of 10;

Accuracy: Recovery values are mentioned in Table 1. They all fell within the range of 98–102%.

Precision: The intra-day precision found on day 1 (*n* = 6) was 0.36%. The inter-day precision investigated over 12 sample solutions performed on two different days was 0.42%. This complied with the RSD requirement of no more than 2%.

### 3.3. Stability of Flucloxacillin in Portable Elastomeric Infusion Pumps at 33 °C for 24 h

The experiment was performed on Floxapen, batch 1P49HK. The initial concentrations of FLU in four PEIPs ranged from 99.9% to 101.5%, with an RSD value of 0.5%. The amount of collected solution at different time points ranged from 31.9 g to 47.5 g, equivalent to 31.2 mL to 46.5 mL when taking the density of the solution (1.0197 mg/mL) into account. This means that the flow rate of the pumps ranged from 7.8 mL/h to 11.6 mL/h. The maximum amount of residual was 3.9 g, equivalent to 3.8 mL, accounting for approximately 1.5% of the total solution (corresponding to 180 mg FLU) that was left in the pump after 24 h.

The pH of the collected solutions was measured in quadruplicate using four PEIPs. Differences between them were negligible, as shown in Table 2. For all four PEIPs, the pH gradually decreased over 24 h to 4.7. The decrease in pH can be explained by the formation of acidic compounds due to the opening of the beta-lactam ring [14].

The concentration of FLU over 24 h is shown in Table 3. In none of the PEIPs did the concentration drop below 92% compared to the initial concentration after 24 h.

No large impurity peaks could be noticed in the chromatograms obtained from freshly prepared sample solutions (example shown in Figure 2). Meanwhile, several impurity peaks could be distinguished in chromatograms obtained with sample solutions collected from the PIEPs that were stored in the oven at 33 °C after 24 h (example shown in Figure 3). FLU can be considered stable under the conditions tested, as degradation was limited to a maximum of 8% (Table 3). Thus, the concentration of FLU remained above the lower acceptance limit of 90%.

In contrast, some publications reported poor stability for FLU in 0.9% NaCl when stored above 31 °C [10,11,12]. However, experimental conditions were different. Carroll [10] reported that a 12 g/240 mL solution in 0.9% NaCl was stable in the refrigerator for 6 days, while To et al. [11] found a loss of about 9% when a 5% solution in 0.9% NaCl was stored in the refrigerator for 14 days. When the solution was stored in the refrigerator for 6 days and then 24 h at 31 °C, the loss of FLU was limited to 6%. However, when the solution was stored in the refrigerator for 6 days and then 17 h at 31 °C and 7 h at 37 °C, the loss was 13% [10]. To et al. [11] even mentioned a loss of 41% when the solution was stored for 6 days in the refrigerator followed by 24 h at 37 °C. Upon degradation, the solution became markedly yellow and turbid with a precipitate, which mainly consisted of FLU acid. The pH of the solutions decreased from 5.5 to about 4.5. To counter this, solutions can be buffered [12,13]. Allwood et al. [12] found that degradation was lowest within the pH range of 6–7. FLU prepared in buffer pH 7.0 was much more stable than in an unbuffered solution. However, using a buffer implies more work and costs, taking into account that the final product should also be sterile regarding parenteral administration. Furthermore, buffered diluents are not generally accepted by practitioners worldwide.

The findings from the above publications led to the idea that storage under refrigerator conditions could have a negative impact on the stability of FLU. Moreover, a temperature increase from 31 °C to 37 °C seems to have a considerable impact. Therefore, additional stability studies of FLU for 24 h at 37 °C were conducted, and the results were compared with those obtained at 33 °C. In addition, the stability of FLU for 6 days in the refrigerator followed by 24 h at 37 °C was compared with the stability for 6 days in the refrigerator followed by 24 h at 33 °C.

### 3.4. Stability of Flucloxacillin in Solution at 37 °C for 24 h

At 37 °C, the pH of both solutions gradually decreased from about 6.2 to about 4.5, which was slightly lower than at 33 °C. At the same time, the concentration of FLU decreased considerably to 83% for solution 1 and 81% for solution 2. The detailed results for both solutions are shown in Table 4.

It can be estimated by interpolation of the data in Table 4 that the concentration of FLU reached 90% after 18 h to 20 h and that the degradation rate increased over time with a rather slow degradation during the first 8 h. It was also noticed for both solutions after 24 h storage at 37 °C that their color turned from colorless to markedly yellow.

When the solutions were kept in the oven for some extra time, it was observed that the solutions became turbid with a white precipitate after an extra 0.5 h. The pH of the solutions was further checked every hour for 6 h. It was noticed that the pH remained stable at approximately 4.4, while the concentration of FLU further decreased to 67.3% for solution 1 and 64.1% for solution 2 after 30 h.

Results shown in Section 3.3 indicated that after 24 h at 33 °C, the pH of the solutions reached 4.7, and the concentration of FLU decreased to no less than 92.6%. These values were similar to those at the time point of 16 h when the solutions were stored at 37 °C. When stored longer, the pH decreased to about 4.5, and precipitation was observed. This occurred faster at 37 °C than at 33 °C, which was consistent with the literature data [15], where the authors indicated that high temperature was the main factor contributing to the lack of stability of FLU.

### 3.5. Stability of Flucloxacillin in Solution under Refrigeration Conditions for 6 Days Followed by 24 h in an Oven

The results obtained from each day of storage in the refrigerator and then followed for 24 h at 33 °C are shown in Table 5.

As shown in Table 5, under refrigeration conditions, the pH of the solution decreased considerably from 6.2 to 5.3, while the FLU concentration remained almost unchanged. After 6 days in the refrigerator, a slight decrease in concentration of no more than 1.5% occurred. This result was consistent with the findings of Carroll [10] and To et al. [11], who found that FLU was stable in 0.9% NaCl for 6 days at 2–8 °C. So, although the pH decreased, the concentration remained quite stable. This was consistent with the profiles in Table 2, Table 3 and Table 4, where the pH dropped from the beginning, while the concentration of FLU remained relatively stable during the first 8 h and started to decrease with a delay compared to the decrease in pH. A somewhat similar phenomenon was observed by To et al. [11], where the concentration of FLU remained at approximately 98% after 6 days in the refrigerator but decreased faster between days 7 and 14 to 91% after two weeks.

When stored for 6 days in the refrigerator plus 24 h at 33 °C, the difference in pH between day 0 (pH 4.7) and day 6 (pH 4.5) was rather small (Table 5). However, the decrease in pH over the last 24 h at 33 °C was considerable: about 1.5 units (from 6.2 (PEIP 1) or 6.3 (PEIP 2) to 4.7) on day 0 and 0.9 units (from 5.4 to 4.5 (PEIP 2)) on day 6. Meanwhile, the concentration of FLU dropped substantially over the last 24 h, with a larger drop for solutions that were stored longer in the refrigerator. After 2 days in the refrigerator plus 24 h at 33 °C, the concentration of FLU decreased by approximately 10%. After 6 days in the refrigerator plus 24 h at 33 °C, this loss almost doubled.

The results of 24 h storage at 33 °C on day 0 were similar to the results obtained in Section 3.3. This confirmed once more that FLU was stable for 24 h after preparation and storage at 33 °C.

After 6 days of refrigeration, pH values and concentrations of FLU were quite similar between the two pumps, amounting to about 5.3 and 99%, respectively (Table 5). However, these values became different after 24 h of storage at different temperatures. The results obtained after 6 days in the refrigerator plus 24 h at 37 °C are presented in Table 6. The results obtained after 6 days in the refrigerator plus 24 h at 33 °C are included in Table 7. The results for day 6 in Table 5 correspond to those at time 0 in Table 6 and Table 7 and at 24 h (Table 7).

As could be expected, degradation was more pronounced at 37 °C, but the difference was quite large since the concentration of FLU decreased after 24 h to 70% at 37 °C, which was 13% lower than when the solution was stored at 33 °C. At 16 h, the pH of the solution stored at 37 °C reached 4.5, and the solution had already turned yellow. At 20 h, some white precipitate was observed. The pH of the solution remained almost unchanged after precipitation. Although no precipitate had yet been formed at 33 °C, the color of the solution changed to yellow after 24 h, while the pH was 4.5.

As additional experiments, all solutions used for the stability study from day 0 to day 6 were kept in the oven until a precipitate was formed. When the pH was checked, it was found that the values remained relatively stable between 4.4 and 4.5, no matter how long the solutions were kept after the precipitation started. These results are consistent with those of the references [10,11]. Carroll [10] found that the degradation was faster at 37 °C than at 31 °C. Meanwhile, To et al. [11] showed a severe loss of 30 to 60% after 6 days in the refrigerator and then 24 h at 37 °C, compared to 30% in our study. References [11,12] confirmed that the stability of FLU was pH-dependent, with maximal stability between pH 6 and 7. In addition, the degradation rate of FLU at pH 4.8 is approximately 10 times faster than at pH 5.7 to 6.2 [11]. Therefore, the sooner a sample reaches pH 4.8, the faster its degradation rate. While pH values were not mentioned in reference [10], the initial pH measured in reference [11] was about 5.5, and after 6 days in the refrigerator, the pH decreased to around 5.0. In our experiments, the initial pH was approximately 6.2, going to 5.3 under the same storage conditions. This indicates that the starting conditions for the two studies were not the same and depended on the FLU samples used to prepare the solutions. This difference could be due to the fact that the tested samples were obtained from different manufacturers and varied somewhat in quality. In our study, FLU 2 g vials from Aurobindo were employed, while FLU 1 g vials from Mayne Pharma, Aspen, and Bowmed Ibisqus were used in references [10,11,12], respectively. In the next step, we examined FLU samples from different batches and brands.

### 3.6. Stability of Flucloxacillin from Different Batches and Brands

Out of four additional batches tested, two batches from Aurobindo showed a similar degradation as above with less than 10% loss of FLU after 24 h when stored at 33 °C. The pH of the solutions at time 0 was around 6.2 and decreased to about 4.6. These results are equivalent to those obtained with batch 1P49HK reported in Section 3.3. Another batch from Aurobindo showed a pH value of 5.3 immediately after reconstitution (0 h). At 33 °C, the pH of the solutions dropped to 4.5 after 24 h. The solutions were still clear without precipitation, but they turned noticeably yellow and quickly formed a precipitate after half an hour under the same condition. The concentration of FLU decreased to approximately 91% after 16 h and 82.3% after 24 h storage. The detailed results are shown in Table 8. The sample from Fresenius Kabi showed the same initial pH value of 5.3. However, the degradation process seemed slightly faster since the solution became yellow without precipitate at 23 h and turbid after an additional hour. Only 79% of FLU remained at 24 h. Detailed results are shown in Table 8.

All solutions complied with the pH requirement according to the Ph. Eur. monograph [9], in which the pH is measured in water and should be in the range from 5.0 to 7.0. Although this will not cause problems for FLU solutions that are injected immediately or within a reasonable timeframe, the pH requirement for the indication of 24 h continuous infusion should be stricter. For a solution with a concentration of 50 mg/mL, the pH measured immediately after reconstitution in saline should best be no less than 5.9.

### 3.7. Stability of Flucloxacillin at Different Temperatures

Table 9 gives an overview of the stability of FLU at different temperatures. Following refrigeration, the FLU concentration was almost unchanged, although the pH of the solutions decreased slightly. At RT, all three solutions were stable for 24 h with at least 95% of FLU remaining. At 33 °C, the solution with a higher initial pH of 6.2 was stable, with about 7% of FLU degrading after 24 h. Meanwhile, a lower initial pH of 5.3 led to instability of the solutions with a degradation of approximately 20%. At 37 °C, all three solutions degraded considerably from 20% to 35%. Moreover, the solutions either turned yellow or formed a precipitate. These results were consistent with those obtained in Section 3.5.

## 4. Conclusions

The quality of the product (responsible for the initial pH after reconstitution) and temperature played important roles in the stability of FLU in saline solution.

It could be expected that a higher temperature had a more detrimental effect on the stability of FLU in solution than a lower temperature, but the impact was quite pronounced. Furthermore, storage in the refrigerator can better be limited in time as much as possible since it has a negative influence on the stability of FLU in solution when used later on at higher temperatures. FLU in 0.9% NaCl that obtained an initial pH of 6.1 degraded to less than 85% after 24 h at 37 °C. Meanwhile, at 33 °C (only 4 °C lower), the concentration of FLU remained higher than 92%. After 6 days in the refrigerator, the concentration of FLU remained almost unchanged, amounting to more than 98%, but the pH of the solution gradually decreased by about 0.9 units. After 6 days of refrigeration and then 24 h at 37 °C, a loss of about 30% of FLU was observed. After 6 days of storage in the refrigerator and then 24 h at 33 °C, a loss of 17% was observed, which was less than at 37 °C, but still substantial. Similar results were observed with the products yielding solutions with an initial pH of 5.3. A storage time of 2 days in the refrigerator seems to be the maximum to later reach more than 90% of FLU when kept at 33 °C for 24 h, mimicking continuous administration via PEIPs to the patient.

In summary, our results indicate that FLU can be prepared in saline without buffer and continuously administered for 24 h via PEIPs for OPAT if two concerns are met. Firstly, the temperature of the solutions in the PEIPs should ideally not exceed 33 °C. Secondly, the pH of the FLU solution should not be lower than 5.9 immediately after reconstitution. To our best knowledge, until now, there have been no studies evaluating all these conditions.

## Figures and Tables

**Table 1:** Accuracy of the method.

Level (mg/mL)	Recovery ± RSD, %
0.04	99.6 ± 1.0
0.05	99.4 ± 0.6
0.06	100.4 ± 1.0

RSD, %: relative standard deviation, expressed in percent.

**Table 2:** pH of the collected solutions over 24 h. The experiment was performed in quadruplicate on four portable elastomeric infusion pumps.

Time (h)	PEIP 1	PEIP 2	PEIP 3	PEIP 4
0	6.1	6.1	6.2	6.2
4	5.6	5.6	5.6	5.7
8	5.5	5.4	5.4	5.5
12	5.2	5.1	5.2	5.3
16	5.0	4.9	4.9	5.0
20	4.8	4.8	4.7	4.8
24	4.7	4.7	4.7	4.7

PEIP: portable elastomeric infusion pump.

**Table 3:** Concentration of flucloxacillin (expressed as per cent) over 24 h taking the initial concentration at 0 h as 100%.

Time (h)	PEIP 1	PEIP 2	PEIP 3	PEIP 4
0	100.0	100.0	100.0	100.0
4	99.3	99.9	99.4	99.2
8	98.8	98.7	98.8	99.1
12	98.1	97.8	97.8	98.8
16	97.1	96.8	97.4	97.0
20	95.0	94.8	96.0	96.0
24	93.4	92.6	94.2	94.7

PEIP: portable elastomeric infusion pump.

**Table 4:** pH and flucloxacillin concentration (expressed as percent, taking the initial concentration at 0 h as 100%) obtained for duplicate solutions over 24 h at 37 °C.

Time (h)	Solution 1		Solution 2	
	pH	FLU (%)	pH	FLU (%)
0	6.2	100.0	6.1	100.0
4	5.6	99.6	5.5	99.3
8	5.2	98.8	5.1	98.8
12	5.0	96.5	4.9	96.2
16	4.7	93.8	4.7	93.2
20	4.7	89.8	4.5	87.7
24	4.5	83.2	4.4	80.9

FLU (%): flucloxacillin concentration, expressed as percent.

**Table 5:** pH and flucloxacillin concentration (expressed as percent, taking the initial concentration as 100%) of solutions in 2 PEIPs over 6 days in the refrigerator plus 24 h at 33 °C.

Time (Day)	PEIP 1				PEIP 2			
	Refrigerator		Refrigerator + 24 h at 33 °C		Refrigerator		Refrigerator + 24 h at 33 °C	
	pH	FLU (%)	pH	FLU (%)	pH	FLU (%)	pH	FLU (%)
0	6.2	100.0	4.7	93.1	6.3	100.0	4.7	93.8
1	5.9	100.3	4.6	91.6	6.0	99.6	4.7	92.2
2	5.8	99.8	4.6	90.3	5.9	100.4	4.7	91.0
3	5.7	100.5	4.6	88.4	5.8	99.9	4.6	89.4
4	5.6	99.4	4.5	86.1	5.6	100.2	4.6	86.8
5	5.5	99.8	4.5	84.2	5.6	99.2	4.6	84.7
6	5.3	98.7	(*)	(*)	5.4	99.0	4.5	83.1

(*) this result could not be reported for storage at 33 °C since pump 1 was stored at 37 °C on day 6. FLU (%): flucloxacillin concentration, expressed as percent.

**Table 6:** Stability of flucloxacillin (expressed as percent, taking the initial concentration as 100%) over 24 h at 37 °C after 6 days of refrigeration.

Time (h)	pH	FLU (%)	Visual Check
0	5.3	98.7	Clear and colorless
4	4.9	96.2	Clear and colorless
8	4.7	93.7	Clear and colorless
12	4.6	89.4	Clear and colorless
16	4.5	86.5	Clear and yellow
20	4.5	79.0	Turbid
24	4.5	70.1	Turbid

FLU (%): flucloxacillin concentration, expressed as percent.

**Table 7:** Stability of flucloxacillin (expressed as percent, taking the initial concentration as 100%) over 24 h at 33 °C after 6 days of refrigeration.

Time (h)	pH	FLU (%)	Visual Check
0	5.4	99.0	Clear and colorless
4	5.0	97.8	Clear and colorless
8	4.8	95.2	Clear and colorless
12	4.8	93.5	Clear and colorless
16	4.6	89.4	Clear and colorless
20	4.6	85.7	Clear and colorless
24	4.5	83.1	Clear and yellow

FLU (%): flucloxacillin concentration, expressed as percent.

**Table 8:** Stability of flucloxacillin for 24 h at 33 °C obtained for two different batches and brands. The flucloxacillin content is expressed as percent, taking the initial concentration at 0 h as 100%.

Time (h)	Tests	Aurobindo, 1P50HK	Aurobindo, 2L26HK	Aurobindo, FL0222027A	Fresenius Kabi, 18Z2229
0	pH	6.3	6.2	5.3	5.3
	FLU (%)	100.0	100.0	100.0	100.0
	Clarity Color	Clear Colorless	Clear Colorless	Clear Colorless	Clear Colorless
16	pH	5.0	4.9	4.6	4.6
	FLU (%)	96.8	96.4	90.9	90.7
	Clarity Color	Clear Colorless	Clear Colorless	Clear Colorless	Clear Colorless
24	pH	4.7	4.6	4.5	4.4
	FLU (%)	93.7	92.8	82.3	78.9
	Clarity Color	Clear Colorless	Clear Colorless	Clear Yellow	Turbid

FLU (%): flucloxacillin concentration, expressed as percent.

**Table 9:** Stability of flucloxacillin at different temperatures. The flucloxacillin content is expressed as percent, taking the initial concentration at 0 h as 100%.

Storage Conditions	Tests	Aurobindo 1P50HK	Aurobindo FL0222027A	Fresenius Kabi, 18Z2229
0 h	pH	6.2	5.3	5.3
	FLU (%)	100.0	100.0	100.0
	Clarity Color	Clear Colorless	Clear Colorless	Clear Colorless
24 h refrigeration	pH	5.9	5.2	5.2
	FLU (%)	99.9	100.2	99.4
	Clarity Color	Clear Colorless	Clear Colorless	Clear Colorless
24 h at RT	pH	5.3	4.8	4.8
	FLU (%)	98.6	95.5	94.8
	Clarity Color	Clear Colorless	Clear Colorless	Clear Colorless
24 h at 33 °C	pH	4.7	4.5	4.4
	FLU (%)	93.4	82.3	78.9
	Clarity Color	Clear Colorless	Clear Yellow	Turbid
24 h at 37 °C	pH	4.5	4.4	4.4
	FLU (%)	81.5	65.5	66.9
	Clarity Color	Clear Yellow	Turbid	Turbid

FLU (%): flucloxacillin concentration, expressed as percent.

## Data Availability

Data are contained within this article.
